# Psychological Capital's Impact on Learned Helplessness Among Nursing Postgraduates: A Path Analysis of Dual Mediation Through Professional Identity Dimensions

**DOI:** 10.1002/nop2.70675

**Published:** 2026-07-27

**Authors:** Jiehui Yang, Wenyu Yue, Xiaoqin Ma

**Affiliations:** ^1^ School of Nursing Zhejiang Chinese Medical University Hangzhou China

**Keywords:** academic engagement, learned helplessness, postgraduate students, professional identity, psychological capital, structural equation model

## Abstract

**Aim:**

To examine the relationships among psychological capital, professional identity, academic engagement, and learned helplessness among nursing postgraduates, and to explore the mediating roles of implicit and practical professional identity.

**Design:**

A multicentre cross‐sectional study.

**Methods:**

A total of 280 nursing postgraduates in China were recruited between January and March 2025. Data were collected using validated instruments assessing psychological capital, professional identity, academic engagement, and learned helplessness. Multiple regression analysis, structural equation modelling, and bootstrapping were conducted to examine direct and mediating relationships.

**Results:**

Multiple regression analysis revealed that professional identity (*β* = −0.23), dedication (*β* = −0.20), and optimism (*β* = −0.30) negatively predicted learned helplessness, while graduate study motivation (*β* = 0.10) was a positive predictor (*R^2^* = 0.351, *p* < 0.01). Path analysis revealed two distinct mediation patterns: Model 1 demonstrated complete mediation, where psychological capital was fully mediated through implicit professional identity (*β* = −2.065, *p* < 0.01) and the chain mediation pathway (*β* = −2.245, *p* < 0.05), accounting for 94.78% of the total effect. Model 2 exhibited partial mediation, with significant direct effects (*β* = −1.169, *p* < 0.01, 55.66%) and smaller indirect effects through practical professional identity (*β* = −0.595, *p* < 0.05) and chain mediation (*β* = −0.182, *p* < 0.05).

**Conclusions:**

This study reveals the different ways in which psychological capital is associated with learned helplessness: through the complete mediation of implicit professional identity and through the partial mediation of practical professional identity. These research results indicate that reducing learned helplessness requires comprehensive intervention targeting the psychological processes and behavioural manifestations of professional identity.

**Implications for the Profession and/or Patient Care:**

The findings highlight the importance of strengthening psychological capital and professional identity to reduce learned helplessness among nursing postgraduates. Nursing educators should implement targeted psychological and educational support to enhance academic engagement and foster a more resilient future nursing workforce.

**Abbreviations**
AES: Academic Engagement Scale
COR: Conservation of resources theory
IBM: Identity‐based motivation
I‐PI: Implicit professional identity
LHQ: Learned Helplessness Scale
MDCIT: Multi‐dimensional career identity theory
PCQ‐24: Psychological Capital Questionnaire
PIQ: Professional Identity Scale
P‐PI: Practical professional identity
SCCT: Social cognitive career theory

## 1 Introduction

### 1.1 Background

Postgraduate nursing students, as high‐quality professionals, are integral to the development of the nursing discipline. Nursing graduate education faces various challenges globally, encompassing academic achievement, mental health, career preparation, and scholarly publication (Leslie et al. 2021; Muraraneza et al. 2020). Under the combined influence of multiple factors, nursing postgraduate students often experience significant academic pressure, which may lead to physical and psychological health issues (Jeong and Koh 2021) and elevated dropout rates (Fang and Zhan 2021). In certain regions, nursing postgraduate students' lack of career interest and confidence has resulted in low publication rates (Khatri et al. 2024), while career‐related prejudices continue to impact their job‐seeking processes and career choices (Beggs et al. 2022). These persistent challenges necessitate increased attention from higher education institutions to address the multifaceted issues faced by nursing postgraduate students and foster their academic and professional success.

The ongoing evolution of nursing education models has brought increased focus to nursing postgraduate students' psychological well‐being (Liu, Zhang, et al. 2023). Among various psychological constructs, psychological capital, emerging from positive psychology research, represents a developmental state of positive personality characteristics (Luthans et al. 2006). This form of capital is particularly malleable and expandable, enabling individuals to effectively manage stressors and demonstrate resilience when confronting challenges (Elliott and Fry 2021; Jiang et al. 2023). As a positive psychological resource, it strengthens individuals' capabilities to navigate adversity and achieve their goals (Guo et al. 2021). Conversely, learned helplessness, first identified through experimental studies (Seligman and Maier 1967), describes a state where individuals become passive and lose their drive to change their situations following repeated unsuccessful experiences. This psychological state significantly influences students' cognitive and behavioural regulation, with higher levels of learned helplessness correlating with increased susceptibility to depressive symptoms (Wu and He 2022). Contemporary research has established that these psychological and motivational elements substantially impact academic outcomes (Cobos‐Sanchiz et al. 2022).

Academic engagement and professional identity emerge as two crucial concepts in education that play pivotal roles in students' professional development. Academic engagement encompasses an individual's cognitive, emotional, and participatory investment in academic activities (Schnitzler et al. 2021). Characterized by active participation, persistence, and enthusiasm, it serves as a significant predictor of academic success and professional growth (Guo et al. 2022). Higher levels of academic engagement enable students to overcome obstacles and achieve deeper learning outcomes (Xu et al. 2023). Professional identity, rooted in career development theory, reflects an individual's understanding and internalization of their professional role and aspirations (Batool and Ghayas 2020). While a strong professional identity fosters a sense of purpose and motivation, inadequate professional identity may lead to decreased academic motivation and difficulties in career decision‐making (Huang and Suan 2023). Professional identity can be understood as multidimensional. Implicit professional identity refers to an internalized sense of emotional attachment, value endorsement, and cognitive identification with the nursing profession. In contrast, practical professional identity refers to the external expression of professional roles, behaviours, and active participation in profession‐related tasks. Distinguishing these dimensions may help explain different psychological pathways.

Current evidence suggests significant interconnections between these constructs. Individuals with higher psychological capital, particularly self‐efficacy and hope, demonstrate greater resilience when confronting setbacks and are less likely to develop patterns of learned helplessness (De Hoe and Janssen 2022). Students with stronger psychological capital exhibit better academic engagement and achievement emotions rather than experiencing anxiety and feelings of powerlessness (Kang et al. 2021). Empirical evidence indicates that psychological capital enhances individuals' perception of event controllability and maintains their ability to retain positive expectations and persist through challenges, directly counteracting the formation of learned helplessness (Baratta et al. 2023).

### 1.2 Theoretical Framework

This study is grounded in Self‐Determination Theory (SDT) (Deci and Ryan 2013) and Identity‐Based Motivation Theory (IBM) (Oyserman 2015). SDT explains how psychological capital enhances intrinsic motivation and engagement, while IBM emphasizes the role of identity in shaping behaviour. Together, these frameworks provide a theoretical basis for the proposed mediation model.

### 1.3 Research Questions

This study aims to address the following questions:

1. What are the relationships among psychological capital, professional identity, academic engagement, and learned helplessness?
2. Do professional identity and academic engagement mediate these relationships?
3. Are there differences between implicit and practical professional identity pathways?

### 1.4 Hypotheses

Psychological capital was positioned as an antecedent variable because it reflects relatively stable positive psychological resources. Professional identity was conceptualized as an internal motivational mechanism shaped by such resources. Academic engagement was then considered a behavioural manifestation of motivational status. Learned helplessness was modelled as a negative psychological outcome. Therefore, the proposed serial order follows a resource–identity–behaviour–outcome logic.

*c. Psychological capital negatively* associates *with learned helplessness*.

Psychological capital plays a vital role in academic engagement, with multiple studies confirming positive correlations between its dimensions and engagement levels. Higher psychological capital indicates greater hope and optimism, which are crucial predictors of academic engagement (Rawat and Devi 2023). When students possess elevated levels of psychological resources, they tend to approach academic tasks more positively and demonstrate stronger willingness for learning engagement. These psychological strengths enable students to perceive challenges as opportunities for growth rather than threats (Paula and Dewi 2020). In academic settings, engagement levels have been identified as crucial factors influencing students' psychological responses to academic challenges, where active participation in learning activities helps students develop mastery experiences and internal locus of control (González et al. 2021). Research suggests that sustained academic involvement enables students to build confidence in their ability to associated with learning outcomes, potentially preventing the development of maladaptive psychological patterns (Bırni 2023). Specifically, when students actively engage in their learning process, they are more likely to maintain a sense of control and develop effective coping strategies when facing academic difficulties. Based on this, we make hypothesis:

*a1. Psychological capital positively associated with academic engagement*.

*b1. Academic engagement negatively associated with learned helplessness*.

Studies exploring professional identity development have revealed its intricate connections with psychological resources and psychological adaptation. The development of positive professional identity relies on higher levels of self‐efficacy and resilience, contributing to clearer professional self‐conception and stronger career commitment (Zhen and Liu 2023). Students with sufficient psychological capital tend to form more positive professional attitudes and demonstrate greater confidence in their career choices (Liu, Han, et al. 2023). Professional identity development also plays a crucial role in students' psychological adaptation to academic challenges. Specifically, strong professional identity enhances the ability to overcome learning difficulties and maintain motivation during challenging periods (Tao and Tien 2024). When students have a clear understanding of their professional role and future career path, they are more likely to maintain adaptive responses to academic setbacks and less prone to developing feelings of helplessness (Adăscăliţei 2020). Based on this, we make hypothesis:

*a2. Psychological capital is positively associated with professional identity*.

*b2. Professional identity negatively associated with learned helplessness*.

Students with stronger professional identity typically demonstrate higher levels of academic engagement in their learning process. When students develop a clear understanding of their future professional role, they often exhibit increased motivation to acquire professional knowledge and skills (Chen et al. 2023). Students demonstrate greater enthusiasm in clinical practice and theoretical learning when they perceive coursework as directly relevant to their future career goals, reflecting deeper engagement in their professional preparation (Morgan Jr and Osborn 2025; Zhang and Tu 2023). Based on this evidence and theoretical framework, we make hypothesis:

*a3. Professional identity positively associated with academic engagement*.

**FIGURE 1:**
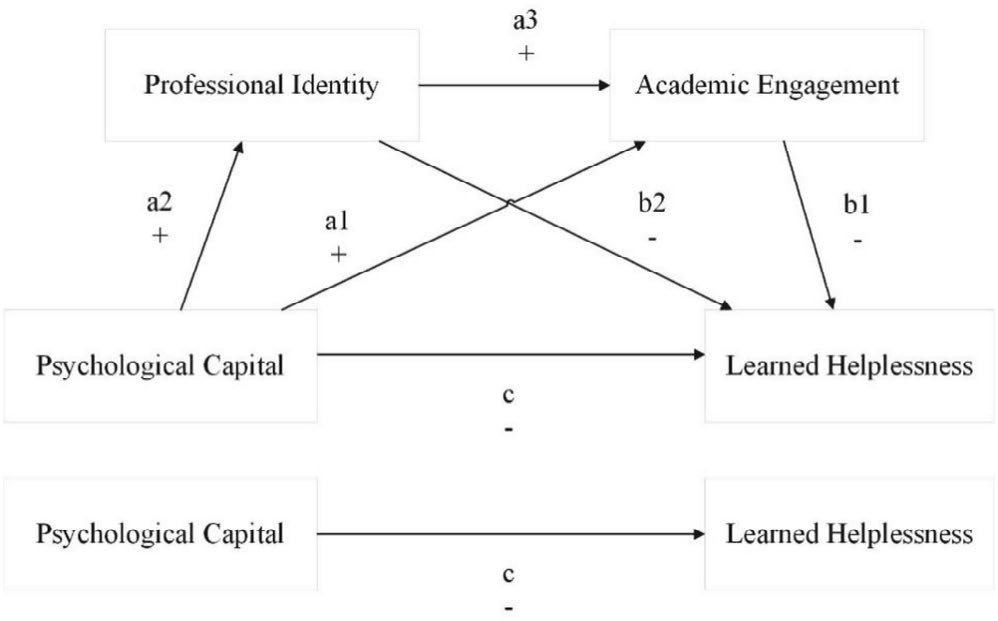
Model hypothesis diagram.

Building on these, the model hypothesis is shown in Figure 1. This study aims to: (1) Examine the current status of psychological capital, professional identity, academic engagement, and learned helplessness among nursing postgraduate students. (2) Identify key demographic and academic factors influencing these constructs. (3) Validate the chain mediating model and explore the deeper mechanisms through path analysis.

Through path analysis, this study seeks to verify the chain mediating effect and clarify the mechanisms through which psychological capital is associated with learned helplessness, with findings expected to inform targeted interventions for enhancing nursing postgraduate students' psychological and academic outcomes.

## 2 Methods

### 2.1 Participants, Design and Setting

**FIGURE 2:**
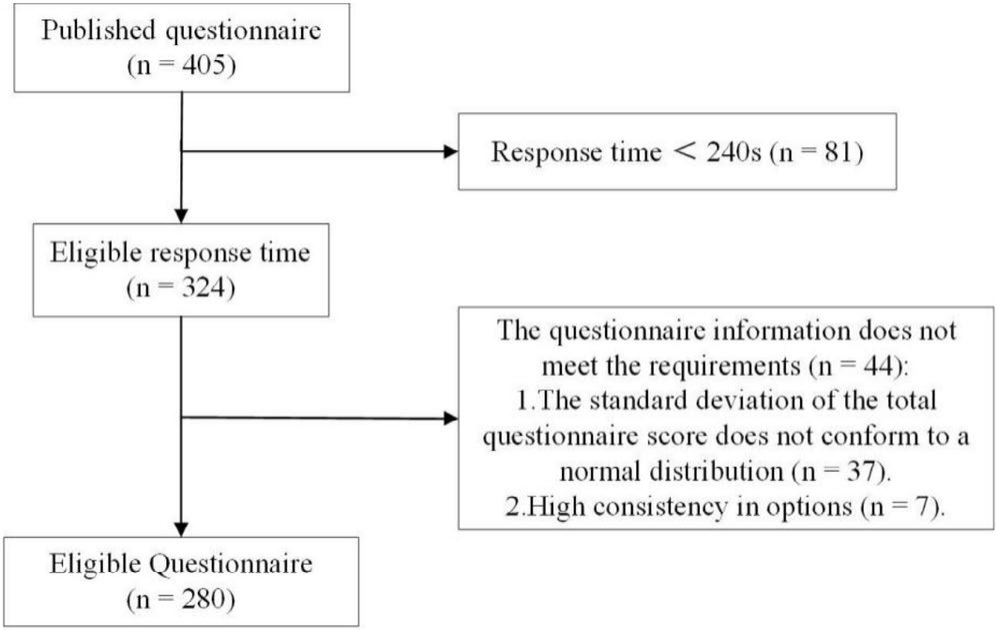
The process of data exclusion.

This study aimed to investigate the relationships among professional identity, academic emotions, and learning engagement in nursing students through structural equation modelling. A cross‐sectional survey design was employed, targeting nursing postgraduates from multiple medical schools across various provinces in China. Due to this design, causal relationships cannot be inferred. Data were collected in January 2025 through an online platform (Questionnaire Star). A convenience sampling method was used, which may limit generalizability. Inclusion criteria were: (1) full‐time nursing postgraduates in bachelor's programs. (2) Ability to complete the survey independently. (3) Voluntary participation with informed consent. Exclusion criteria included students in graduate/diploma programs and those without current academic enrollment. Of 405 distributed questionnaires, 280 valid responses were included (response rate: 69.1%). Invalid responses were excluded based on completion time (less than 240 s, determined through pilot testing) or patterned answers. The participant selection process is detailed in Figure 2 and the STROBE checklist for cross‐sectional studies detailing the reporting of each methodological component is available in Supporting Information.

### 2.2 Measures

This study examined four primary variables: academic engagement, learned helplessness, psychological capital, and professional identity.

#### 2.2.1 General Information Questionnaire

The questionnaire designed by researchers collected demographic and professional data, including academic, personal, and family‐related variables that previous studies have identified as potential influencing factors in nursing education outcomes. These variables included grade, gender, student leadership experience, major choice motivation, and educational background factors.

#### 2.2.2 Psychological Capital Questionnaire (PCQ‐24)

The PCQ‐24 was selected for its established validity in measuring psychological resources in educational settings and its specific validation among Chinese nursing students. This scale, developed by Luthans et al. 8 and adapted for Chinese nursing students by Li (Luthans et al. 2008), comprehensively assesses self‐efficacy, optimism, hope, and resilience. The 6‐point Likert scale ranges from 1 (“strongly disagree”) to 6 (“strongly agree”), with reverse scoring for three items. Total scores range from 24 to 144, with higher scores indicating higher levels of psychological capital. The Cronbach's alpha coefficient was 0.912.

#### 2.2.3 Learned Helplessness Scale (LHQ)

The LHQ was chosen for its specific focus on academic‐related helplessness and its proven reliability in nursing education research. Developed by Seligman (Hiroto and Seligman 1975) and adapted for Chinese nursing students by Wu et al. (Wu et al. 2009), it effectively captures both helplessness (13 items) and despair (5 items) dimensions. Items are scored on a 5‐point Likert scale, with higher scores indicating stronger tendencies towards learned helplessness. The Cronbach's alpha coefficient was 0.930.

#### 2.2.4 Academic Engagement Scale (AES)

The AES was selected for its robust theoretical foundation and widespread use in higher education research. Developed by Schaufeli et al. (Schaufeli et al. 2003) and translated into Chinese by Li et al. (Li and Huang 2010), this scale effectively measures the multidimensional nature of academic engagement through vigour (6 items), dedication (5 items), and absorption (6 items). Items are scored on a 5‐point Likert scale, with higher scores indicating higher engagement levels. The Cronbach's alpha was 0.913.

#### 2.2.5 Professional Identity Scale (PIQ)

The PIQ was chosen for its comprehensive coverage of professional identity dimensions and specific validation in Chinese nursing education contexts. Developed by Qin (Qin 2009), this scale uniquely captures both internal and external aspects of professional identity through four dimensions: cognitive identity (5 items), emotional identity (8 items), behavioural identity (6 items), and adaptive identity (4 items). Items use a 5‐point Likert scale, with higher scores indicating stronger professional identity. The Cronbach's alpha coefficient was 0.97.

In this study, inspired by Chen et al.'s (2022) conceptualization of explicit and implicit professional identity among teacher education students (Chena et al. 2022), cognitive identity and emotional identity are categorized as Implicit Professional Identity (I‐PI), representing the internal psychological aspects of professional identification. Behavioural identity and adaptive identity are categorized as Practical Professional Identity (P‐PI), reflecting the external behavioural manifestations of professional role integration. For consistency, subsequent tables and figures use these abbreviated terms to represent the reorganized dimensions.

### 2.3 Sample Size

The sample size was estimated following Kendall's recommendation of 5–10 respondents per variable (Zhou et al. 2020). With 13 variables examined in this study, a minimum of 156 valid responses was required, considering a 20% margin to account for non‐responses or invalid data. The inclusion of 280 valid responses ensured sufficient statistical power and robustness in the findings.

### 2.4 Data Collection

Data were collected from January 2025, using the Questionnaire Star platform to ensure efficiency and accessibility. Participants were provided with detailed instructions before completing the survey, including the study purpose, confidentiality assurance, and response guidelines. To maintain data quality, several measures were implemented: (1) IP addresses were restricted to single submissions. (2) Surveys had to be completed within a one‐hour time frame. (3) Two researchers independently reviewed all collected data, applying strict exclusion criteria for questionnaires completed in less than 240 s or exhibiting patterned responses.

### 2.5 Data Analysis

Data were analysed using SPSS 26.0 and Amos 24.0 to provide comprehensive insights. Descriptive statistics were used to summarize sample characteristics. Independent *t*‐tests, ANOVA, and post hoc analyses were conducted to compare groups, while Pearson correlation and stepwise linear regression identified significant predictors of learned helplessness. Structural equation modelling (SEM) was employed in Amos to test hypothesized relationships among variables, with path analysis highlighting direct and indirect effects. Bootstrapping with 5000 random samples was used to evaluate mediation effects, and a 95% bias‐corrected confidence interval excluding zero was considered evidence of significant mediation. Missing data were minimal and addressed using listwise deletion. All tests were two‐tailed, with significance set at *p* < 0.05. Confirmatory factor analysis (CFA) was conducted to evaluate the measurement model. All factor loadings exceeded 0.60. Composite reliability (CR) ranged from 0.82 to 0.94, and average variance extracted (AVE) ranged from 0.51 to 0.73, indicating good convergent validity. Discriminant validity was supported as the square root of AVE for each construct exceeded inter‐construct correlations. The structural model demonstrated acceptable fit: χ^2^/df = 2.31, CFI = 0.93, TLI = 0.91, RMSEA = 0.069, SRMR = 0.048.

## 3 Results

### 3.1 Common Method Bias

Harman's single‐factor test showed the first factor explained 28.23% variance (< 40%), indicating no serious bias.

### 3.2 Participants' Sociodemographic Characteristics and Univariate Analysis of Learned Helplessness

Among 280 participants, significant differences in learned helplessness were found in age (*p* = 0.04), study mode (*p* < 0.01), professional identity (*p* < 0.01), work experience (*p* = 0.02), reason for pursuing studies (*p* = 0.02), and supervisor's communication style (*p* < 0.01). Detailed results are presented in Table 1 and the results of post hoc tests examining differences in innovative behaviour scores across demographic variables are provided in Supporting Information.

**TABLE 1:** Demographic data and univariate analysis of postgraduate nursing students' learned helplessness (*n* = 280).

Variable	Frequency (%)	Learned helplessness Scale	t/F	*p*
Gender				
Male	18	46.67 ± 13.39	0.78	0.44
Female	262	44.26 ± 12.73		
Age				
21–25 years old	133	46.16 ± 12.69	2.86	0.04*
26–30 years old	50	45.20 ± 13.30		
31–35 years old	54	42.80 ± 12.29		
≥ 35 years old	43	40.12 ± 12.20		
Degree type				
Professional	153	45.56 ± 12.77	1.66	0.1
Academic	127	43.02 ± 12.67		
Study mode				
Full‐time	183	45.77 ± 12.61	2.46	< 0.01**
Part‐time	97	41.86 ± 12.71		
Year of study				
First year graduate student	102	43.62 ± 12.22	2.52	0.06
Second year graduate student	54	45.15 ± 13.44		
Third year graduate student	48	48.50 ± 12.13		
Three years or more	76	42.37 ± 12.99		
First academic degree				
Associate degree	35	43.23 ± 12.67	1.75	0.18
Associate to bachelor's degree	21	39.86 ± 12.44		
Bachelor's degree	224	45.02 ± 12.76		
Type of university attended				
Regular university	229	43.70 ± 12.62	−1.99	0.05*
985 or 211 or double first‐class university	51	47.61 ± 13.03		
Student leadership experience				
Yes	168	43.58 ± 12.43	−1.33	0.18
No	112	45.65 ± 13.20		
Only child				
Yes	85	43.45 ± 13.60	−0.83	0.41
No	195	44.83 ± 12.39		
Professional identity				
Low	26	50.88 ± 9.06	16.76	< 0.01**
Average	169	46.46 ± 12.27		
High	85	38.36 ± 12.53		
Family residence				
Rural area	123	44.70 ± 12.74	0.18	0.84
Town	72	43.64 ± 12.54		
City	85	44.65 ± 13.09		
Work experience				
None	128	46.31 ± 12.06	3.2	0.02*
≤ 2 years	30	41.27 ± 14.28		
2–5 years	13	49.54 ± 12.57		
≥ 5 years	109	42.43 ± 12.77		
Reason for pursuing graduate studies				
Voluntary	142	42.50 ± 12.17	3.46	0.02*
Employment or promotion pressure	67	47.13 ± 12.92		
Recommendation from others	16	50.69 ± 12.50		
Improve research skills	55	44.20 ± 13.27		
Supervisor's communication style with students				
Tends to encourage	184	42.93 ± 12.60	5.45	< 0.01**
Tends to criticize	5	57.40 ± 10.43		
Tends to supervise	91	46.69 ± 12.58		
Supervisor's requirements				
Strict	44	44.93 ± 13.68	0.92	0.43
Relatively strict	155	43.37 ± 13.16		
Relatively lenient	63	45.79 ± 11.13		
Lenient	18	47.28 ± 12.29		

* *p* < 0.05.

** *p* < 0.01.

### 3.3 Scores of Academic Engagement, Learned Helplessness, Psychological Capital, and Professional Identity

The descriptive analysis showed that the total scores were 103.86 ± 13.40 for psychological capital, 44.41 ± 12.76 for learned helplessness, 54.89 ± 8.82 for academic engagement, and 88.12 ± 12.77 for professional identity. The specific scores of each dimension are shown in Table 2.

**TABLE 2:** Descriptive analysis of academic engagement, learned helplessness, psychological capital, and professional identity (*n* = 280).

Scale	Dimension	M ± SD
Psychological Capital Questionnaire	Self‐efficacy	26.97 ± 3.95
	Hope	25.76 ± 4.06
	Resilience	25.82 ± 3.66
	Optimism	25.31 ± 4.07
	Total score	103.86 ± 13.40
Learned Helplessness Scale	Helplessness	35.06 ± 10.41
	Despair	9.35 ± 3.53
	Total score	44.41 ± 12.76
Academic Engagement Scale	Vigour	18.11 ± 3.56
	Dedication	17.34 ± 3.07
	Absorption	19.43 ± 3.58
	Total score	54.89 ± 8.82
Professional Identity Scale	Cognitive identity	20.55 ± 2.77
	Emotional identity	29.43 ± 6.56
	Behavioural identity	23.76 ± 3.61
	Adaptive identity	14.39 ± 2.93
	Total score	88.12 ± 12.77

### 3.4 Correlations Among Academic Engagement, Learned Helplessness, Psychological Capital, and Professional Identity

All main variables demonstrated significant correlations with each other (*p* < 0.01). Specifically, psychological capital showed strong positive correlations with academic engagement (*r* = 0.611) and professional identity (*r* = 0.628), while displaying a significant negative correlation with learned helplessness (*r* = −0.531). Academic engagement was positively correlated with professional identity (*r* = 0.575) and negatively correlated with learned helplessness (*r* = −0.439). Detailed correlations between dimensions are presented in Table 3.

**TABLE 3:** Correlation analysis of academic engagement, learned helplessness, psychological capital, and professional identity (*n* = 280).

Pearson correlation analysis	1	2	3	4	5	6	7	8	9	10	11	12	13	14	15	16	17
1.Self‐Efficacy	1	0.74**	0.625**	0.561**	0.861**	−0.421**	−0.332**	−0.436**	0.524**	0.486**	0.389**	0.539**	0.332**	0.418**	0.412**	0.432**	0.502**
2.Hope	0.74**	1	0.698**	0.602**	0.895**	−0.452**	−0.313**	−0.456**	0.575**	0.474**	0.424**	0.569**	0.298**	0.509**	0.447**	0.511**	0.57**
3.Resilience	0.625**	0.698**	1	0.565**	0.841**	−0.399**	−0.286**	−0.405**	0.541**	0.451**	0.373**	0.527**	0.288**	0.448**	0.44**	0.477**	0.527**
4.Optimism	0.561**	0.602**	0.565**	1	0.806**	−0.494**	−0.367**	−0.505**	0.425**	0.422**	0.318**	0.448**	0.34**	0.457**	0.441**	0.447**	0.536**
5. Psychological Capital Questionnaire Scores	0.861**	0.895**	0.841**	0.806**	1	−0.521**	−0.383**	−0.531**	0.606**	0.539**	0.442**	0.611**	0.371**	0.539**	0.511**	0.548**	0.628**
6.Helplessness	−0.421**	−0.452**	−0.399**	−0.494**	−0.521**	1	0.569**	0.974**	−0.406**	−0.41**	−0.274**	−0.418**	−0.252**	−0.428**	−0.351**	−0.461**	−0.48**
7.Despair	−0.332**	−0.313**	−0.286**	−0.367**	−0.383**	0.569**	1	0.741**	−0.306**	−0.385**	−0.235**	−0.353**	−0.195**	−0.327**	−0.281**	−0.287**	−0.356**
8.Learned Helplessness Scale Scores	−0.436**	−0.456**	−0.405**	−0.505**	−0.531**	0.974**	0.741**	1	−0.416**	−0.441**	−0.289**	−0.439**	−0.26**	−0.44**	−0.365**	−0.456**	−0.49**
9.Vigour	0.524**	0.575**	0.541**	0.425**	0.606**	−0.406**	−0.306**	−0.416**	1	0.588**	0.637**	0.867**	0.168**	0.421**	0.489**	0.525**	0.512**
10.Dedication	0.486**	0.474**	0.451**	0.422**	0.539**	−0.41**	−0.385**	−0.441**	0.588**	1	0.627**	0.84**	0.328**	0.414**	0.459**	0.467**	0.521**
11.Absorption	0.389**	0.424**	0.373**	0.318**	0.442**	−0.274**	−0.235**	−0.289**	0.637**	0.627**	1	0.881**	0.226**	0.353**	0.455**	0.444**	0.461**
12.Academic Engagement Scale Scores	0.539**	0.569**	0.527**	0.448**	0.611**	−0.418**	−0.353**	−0.439**	0.867**	0.84**	0.881**	1	0.274**	0.458**	0.541**	0.555**	0.575**
13.Cognitive Identity	0.332**	0.298**	0.288**	0.34**	0.371**	−0.252**	−0.195**	−0.26**	0.168**	0.328**	0.226**	0.274**	1	0.282**	0.401**	0.29**	0.542**
14.Emotional Identity	0.418**	0.509**	0.448**	0.457**	0.539**	−0.428**	−0.327**	−0.44**	0.421**	0.414**	0.353**	0.458**	0.282**	1	0.59**	0.681**	0.898**
15.Behavioural Identity	0.412**	0.447**	0.44**	0.441**	0.511**	−0.351**	−0.281**	−0.365**	0.489**	0.459**	0.455**	0.541**	0.401**	0.59**	1	0.641**	0.82**
16.Adaptive Identity	0.432**	0.511**	0.477**	0.447**	0.548**	−0.461**	−0.287**	−0.456**	0.525**	0.467**	0.444**	0.555**	0.29**	0.681**	0.641**	1	0.824**
17.Professional Identity Scale Scores	0.502**	0.57**	0.527**	0.536**	0.628**	−0.48**	−0.356**	−0.49**	0.512**	0.521**	0.461**	0.575**	0.542**	0.898**	0.82**	0.824**	1

* *p* < 0.05.

** *p* < 0.01.

### 3.5 Multiple Linear Regression of Learned Helplessness in Postgraduate Nursing Students

Stepwise linear regression analysis identified four significant predictors of learned helplessness (*F* = 38.773, *p* < 0.01), explaining 35.1% of the total variance (adjusted *R^2^* = 0.351). Professional identity (*β* = −0.23, *p* < 0.01), dedication (*β* = −0.20, *p* < 0.01), and optimism (*β* = −0.30, *p* < 0.01) were negative predictors, while reason for pursuing graduate studies (*β* = 0.10, *p* < 0.05) was a positive predictor. All variance inflation factors (VIF) were less than 2, indicating no multicollinearity. See Table 4 for details.

**TABLE 4:** Stepwise linear regression of learned helplessness in postgraduate nursing students (*n* = 280).

Items	Regression coefficient	Standard error	Standardized regression coefficient	t	*p*	Covariance tolerance	VIF
Constant	100.55	4.88		20.62	< 0.01**		
Professional Identity Scale Scores	−0.23	0.06	−0.23	−3.74	< 0.01**	0.61	1.65
Dedication	−0.84	0.24	−0.20	−3.51	< 0.01**	0.70	1.43
Optimism	−0.92	0.18	−0.30	−5.05	< 0.01**	0.68	1.46
Reason for pursuing graduate studies	1.15	0.53	0.10	2.16	0.03*	0.99	1.01

*Note:* *R* ^2^ = 0.361, adjust *R* ^2^ = 0.351, F = 38.773, *p* < 0.00.

* *p* < 0.05.

** *p* < 0.01.

### 3.6 A Path Analysis of Academic Engagement, Learned Helplessness, Psychological Capital, and Professional Identity

Overall, hypotheses [Link], [Link], [Link], [Link], [Link], [Link] were supported, except for the direct effect in Model 1, which was not statistically significant.

#### 3.6.1 The Chain Mediating Effect of Implicit Professional Identity and Academic Engagement Between Psychological Capital and Learned Helplessness

**FIGURE 3:**
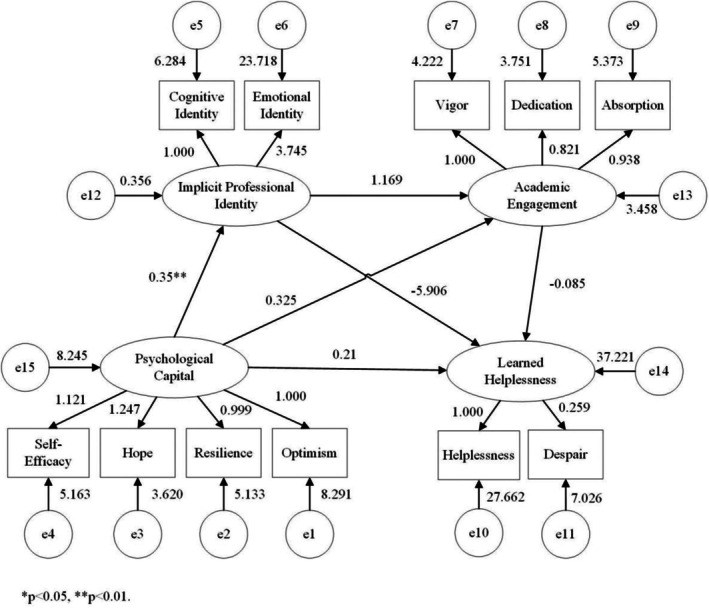
Path analysis of psychological capital through implicit professional identity on academic engagement (*n* = 280).

Path analysis revealed that psychological capital positively predicted implicit professional identity (*β* = 0.35, *p* < 0.01). The path coefficient from psychological capital to learned helplessness was 0.21, while implicit professional identity to academic engagement showed a coefficient of 1.169, and academic engagement to learned helplessness was −0.085 (Figure 3).

#### 3.6.2 The Chain Mediating Effect of Practical Professional Identity and Academic Engagement Between Psychological Capital and Learned Helplessness

**FIGURE 4:**
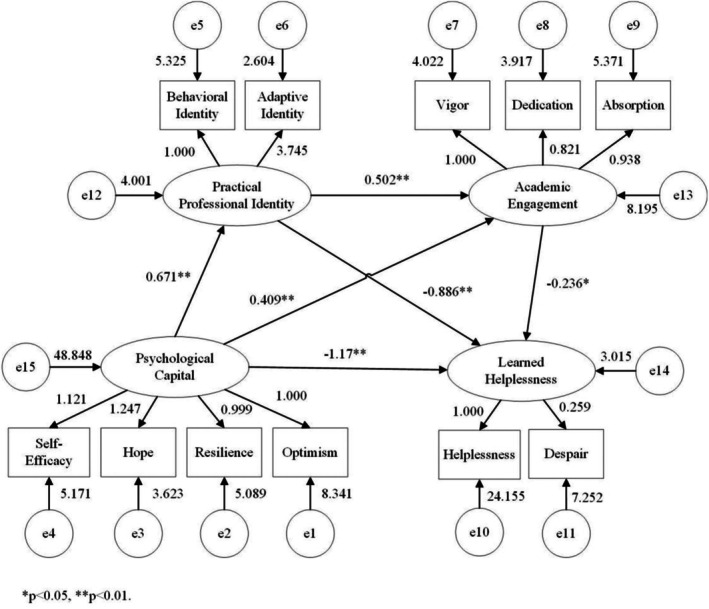
Path analysis of psychological capital through practical professional identity on academic engagement (*n* = 280).

Path analysis showed significant relationships among all variables: psychological capital to practical professional identity (*β* = 0.671, *p* < 0.01), to academic engagement (*β* = 0.409, *p* < 0.01), and to learned helplessness (*β* = −1.17, *p* < 0.01). Practical professional identity significantly predicted both academic engagement (*β* = 0.502, *p* < 0.01) and learned helplessness (*β* = −0.886, *p* < 0.01). Academic engagement negatively predicted learned helplessness (*β* = −0.236, *p* < 0.05) (Figure 4).

### 3.7 Bootstrap Test

While the direct effect of psychological capital on learned helplessness showed no significance (Figure 3), the Bootstrap analysis results (Table 5) demonstrated significant indirect effects through implicit professional identity (*β* = −2.065, *p* < 0.01) and the chain mediation of “implicit professional identity‐academic engagement” (*β* = −2.245, *p* < 0.05). In Model 2, psychological capital showed significant mediating effects through practical professional identity (*β* = −0.595, *p* < 0.05) and the “practical professional identity‐academic engagement” path (*β* = −0.182, *p* < 0.05).

**TABLE 5:** Bootstrap test of mediation effect (*n* = 280).

Model	Pathway	SE	Estimate	Lower	Upper	*p*	Proportion
Model 1	Psychological capital → Academic engagement → Learned helplessness	0.914	−0.028	−0.681	0.433	0.95	0.62%
	Psychological capital → Implicit professional identity → Learned helplessness	3.328	−2.065	−18.154	−0.525	< 0.01**	45.41%
	Psychological capital → Implicit professional identity → Academic engagement → Learned helplessness	59.425	−2.245	−128.417	−0.645	0.02*	49.37%
	Direct effect	5.123	0.209	−9.832	10.25	0.967	4.60%
	Total effect	58.253	−4.129	−79.416	−2.311	< 0.01**	100.00%
Model 2	Psychological capital → Academic engagement → Learned helplessness	0.193	−0.097	−0.478	0.295	0.549	4.35%
	Psychological capital → Practical professional identity → Learned helplessness	0.29	−0.595	−1.205	−0.015	0.04*	31.84%
	Psychological capital → Practical professional identity → Academic engagement → Learned helplessness	0.117	−0.182	−0.498	−0.012	0.04*	8.15%
	Direct effect	0.277	−1.169	−1.712	−0.626	< 0.01**	55.66%
	Total effect	0.355	−2.043	−2.783	−1.391	< 0.01**	100.00%

* *p* < 0.05.

** *p* < 0.01.

## 4 Discussion

### 4.1 Predictive Factors of Learned Helplessness Among Nursing Postgraduates: A Multiple Regression Analysis

#### 4.1.1 Professional Identity

The stepwise regression analysis revealed that Implicit Professional Identity (I‐PI) and Practical Professional Identity (P‐PI) are significant negative predictors of learned helplessness among nursing postgraduate students. Students with stronger professional identity may demonstrate clearer professional values and career goals, which can enhance resilience when facing academic challenges (Jilili et al. 2024). This finding is consistent with previous studies showing that professional identity supports adaptive coping and psychological well‐being among nursing students (Mei et al. 2022). A stronger professional identity may help nursing postgraduates interpret academic difficulties more positively and reduce vulnerability to helplessness (Zhang et al. 2021). Therefore, educational strategies aimed at strengthening professional identity may contribute to reducing learned helplessness among nursing postgraduates.

#### 4.1.2 Dedication

Dedication was also identified as a significant negative predictor of learned helplessness. Dedication reflects students' enthusiasm and commitment to academic activities and has been associated with positive educational outcomes (Muñoz‐García and Villena‐Martínez 2021). Students with higher dedication may be more likely to remain engaged when facing academic difficulties, thereby reducing feelings of helplessness (Lei 2022). Students with high dedication levels are more likely to view obstacles as growth opportunities rather than insurmountable barriers, demonstrating better emotional regulation mechanisms that enhance their psychological resilience (Liu, Zhong, et al. 2023). Supporting meaningful academic engagement may therefore help reduce learned helplessness among nursing postgraduates (Ji et al. 2024).

#### 4.1.3 Optimism

Optimism is the strongest negative predictor of learned helplessness. This finding is consistent with previous studies suggesting that optimistic individuals are more likely to adopt adaptive coping strategies when encountering challenges (Gordeeva et al. 2020). As an important component of psychological capital, optimism may help students maintain positive expectations and resilience during academic stress (López‐Guerra et al. 2023). Educational programmes aimed at fostering positive coping strategies may help strengthen students' resilience and reduce learned helplessness.

#### 4.1.4 Graduate Study Motivation

Graduate study motivation was positively associated with learned helplessness. Students who pursued graduate education due to external pressures showed higher levels of learned helplessness than those with autonomous motivations. This finding may reflect reduced autonomy and lower intrinsic motivation among students with externally driven educational goals (Howard et al. 2021). This state of limited autonomy and intrinsic motivation typically leads to reduced psychological resilience when facing academic challenges, ultimately increasing their susceptibility to learned helplessness (Jeno et al. 2023). Educational institutions may benefit from providing career counselling and motivational support to help students strengthen autonomous learning motivation (Pineda et al. 2024).

### 4.2 The Mediating Role of Implicit Professional Identity

Model 1 demonstrated that psychological capital was indirectly associated with learned helplessness through implicit professional identity. This finding suggests that professional identity may play an important role in translating psychological resources into adaptive psychological outcomes.

The persistent non‐significance of direct effects even under multiple influencing factors demonstrates the complete mediating role of these pathways in the relationship between psychological capital and learned helplessness.

This finding is consistent with previous studies indicating that professional identity may function as a psychological resource that supports resilience and adaptive coping among nursing students (Oyserman 2015). As a core component of self‐concept, implicit professional identity helps students transform psychological resources like optimism and resilience into self‐awareness, thereby indirectly influencing their behaviour, such as reducing vulnerability to helplessness (Alfrey et al. 2023). When students possess higher psychological capital, they tend to develop a stronger internalized professional self‐concept that serves as a protective shield against feelings of helplessness (Kim 2024). The sequential mediation through implicit professional identity and academic engagement reveals a more complex pathway, which can be explained through Self‐Determination Theory (Deci and Ryan 2013). SDT emphasizes the importance of intrinsic motivation in shaping external behaviours (Ryan and Deci 2020), where implicit professional identity enhances students' competence (e.g., belief in their ability to fulfil professional responsibilities) and relatedness (e.g., connection to their profession) to satisfy psychological needs, while academic engagement represents the behavioural manifestation of this intrinsic motivation. This dynamic process demonstrates how identity‐based construction and behavioural engagement work together to mitigate learned helplessness, revealing a powerful mechanism of psychological resilience.

The absence of a significant direct effect suggests that psychological capital may influence learned helplessness through indirect psychological and behavioural pathways rather than immediate emotional responses (Darvishmotevali and Ali 2020). These findings suggest that nursing education programmes may benefit from strategies that strengthen professional identity and promote meaningful academic engagement to reduce learned helplessness (Howard et al. 2021).

### 4.3 Differential Effects Between Implicit and Practical Professional Identity Models

The stronger association observed in the I‐PI model may reflect the internalized nature of implicit professional identity. Compared with practical professional identity, implicit professional identity may represent deeper cognitive and emotional attachment to the profession, potentially providing greater psychological stability during academic challenges.

This finding is consistent with previous studies suggesting that stronger professional identity is associated with better psychological adaptation among nursing students. Students with stronger internalized professional values may be better able to maintain confidence and resilience when facing academic stress (Van den Broek et al. 2020). Graduate nursing students may rely more heavily on professional identity because postgraduate education often involves prolonged academic and professional challenges (Jarden et al. 2021). While external behaviours can indeed provide short‐term emotional buffering, their impact appears more limited in shaping students' long‐term psychological responses, potentially failing to help them address deeper doubts or feelings of helplessness when challenges arise (Olivier et al. 2020). Consequently, nursing graduate students are more likely to benefit from deeper, identity‐centred development rather than merely deriving emotional value from behavioural competencies (Malandraki 2022). Research indicates that current nursing graduate students exhibit relatively low professional identity (Deng et al. 2020). Therefore, the internalization of professional roles and values becomes increasingly crucial, as this pathway enables students to develop a more robust psychological foundation (López‐Íñiguez et al. 2022). These findings suggest that implicit professional identity may play a stronger protective role against learned helplessness than practical professional identity.

The non‐significant mediating effect of academic engagement suggests that engagement alone may not sufficiently explain learned helplessness among nursing postgraduates. One possible explanation is that postgraduate education is highly research‐oriented, limiting the influence of engagement on psychological outcomes (Xiong et al. 2024). Professional identity may therefore play a more central role than academic engagement in supporting psychological adjustment among nursing postgraduates (Yu et al. 2021).

These findings suggest that graduate programmes should strengthen professional identity development alongside practical training to reduce learned helplessness.

### 4.4 Different Mediation Mechanisms of Professional Identity

The two professional identity models demonstrated different mediation patterns in the relationship between psychological capital and learned helplessness. These distinct mediation patterns suggest fundamentally different mechanisms through which the two dimensions of professional identity are associated with learned helplessness.

One possible explanation is that implicit professional identity reflects internalised beliefs and values, whereas practical professional identity is more closely related to behavioural adaptation (Porfeli et al. 2011). Within this process, academic engagement serves as a crucial link in the chain mediation by transforming intrinsic motivation into active participation. This dual‐mediation pattern reflects the layered and reflexive nature of implicit identity, which guides motivation and behaviour through gradual identity activation and situational interaction (Walder et al. 2022).

In contrast, practical professional identity demonstrated a stronger direct association, suggesting that behavioural competencies may directly support students in coping with academic stress (Labrague 2021). Practical professional identity may help students apply psychological resources through skill performance and role adaptation (Walder et al. 2022). This finding is consistent with previous studies emphasizing the role of psychological resources and professional identity in psychological adjustment (Zola et al. 2022).

Educational institutions may benefit from combining psychological support with professional development activities to strengthen both identity formation and practical competencies (Mohd Rasdi and Ahrari 2020).

### 4.5 Limitations and Prospects

This study has several limitations. First, the cross‐sectional design limits the ability to establish causal relationships, and the mediation results should be interpreted with caution. Second, the use of convenience sampling may restrict the generalizability of the findings. Third, all variables were measured using self‐reported instruments, which may introduce response bias despite the acceptable results of common method bias testing.

## 5 Conclusion

This study reveals distinct pathways through which psychological capital influences learned helplessness among nursing postgraduates: complete mediation through implicit professional identity versus partial mediation via practical professional identity. The findings highlight the crucial role of internalised professional values in buffering against learned helplessness, with implicit professional identity showing stronger protective effects compared to behavioural manifestations. These results suggest that reducing learned helplessness requires comprehensive interventions targeting both psychological processes and behavioural aspects of professional identity. Educational institutions should implement integrated support programmes that enhance psychological capital while fostering professional identity development, particularly emphasising the internalisation of professional values and roles. Future research should validate these findings across different cultural contexts and examine the long‐term effectiveness of targeted interventions based on the dual‐pathway model.

## Author Contributions

Jiehui Yang: conceptualization, methodology, formal analysis, investigation, writing – original draft, writing – review and editing, data collection. Wenyu Yue: data collection, writing – original draft. Xiaoqin Ma: Conceptualization, methodology, investigation, supervision, project administration, writing – review and editing.

## Funding

The authors have nothing to report.

## Ethics Statement

This study was conducted in accordance with the Declaration of Helsinki and followed the STROBE guidelines for reporting observational studies. Ethics approval was obtained from the Ethics Committee of Zhejiang Chinese Medical University (Approval number: 20241213‐4).

## Consent

All participants provided signed informed consent.

### Patient or Public Contribution

This observational study did not directly involve patients, service users, caregivers, or members of the public in its design, conduct, or reporting. All participants were enrolled nursing postgraduate students who voluntarily completed anonymous online questionnaires. The research focused on psychological mechanisms within this specific student group and did not include intervention in or data collection from patient populations. Findings are therefore primarily relevant to postgraduate nursing education and student support systems, though they may indirectly inform future improvements in nursing practice and public health through educational translation.

## Conflicts of Interest

The authors declare no conflicts of interest.

## Supporting information


**Data S1:** STROBE Statement—Checklist of items that should be included in reports of *cross‐sectional studies*.

**Data S2:** One‐factor test post hoc test.

## Data Availability

The datasets generated and/or analysed during the current study are not publicly available due to institutional privacy policies and ethics considerations regarding participant confidentiality but are available from the corresponding author on reasonable request subject to data sharing agreement.
