# Supplementary material for: Psychological Capital's Impact on Learned Helplessness Among Nursing Postgraduates: A Path Analysis of Dual Mediation Through Professional Identity Dimensions
Source: Nurs Open. 2026 Jul 27;13(7):e70675. doi: 10.1002/nop2.70675 (PMC13403486; doi:10.1002/nop2.70675)
Supplement: Supplementary file 2 — Data S2: One‐factor test post hoc test. [file NOP2-13-e70675-s001.docx]

**One-factor test post hoc test**

**Outcome:** Learned Helplessness Scale Scores

1.Professional identity

Method: LSD

| (I) Professional identity | (J) Professional identity | Mean Difference (I-J) | Std. error | P value | 95% Confidence interval | |
| --- | --- | --- | --- | --- | --- | --- |
|  |  |  |  |  | Lower bound | Upper bound |
| Low | Average | 4.43 | 2.55 | 0.08 | -0.59 | 9.45 |
|  | High | 12.52 | 2.71 | ＜0.01** | 7.18 | 17.86 |
| Average | Low | -4.43 | 2.55 | 0.08 | -9.45 | 0.59 |
|  | High | 8.09 | 1.61 | ＜0.01** | 4.92 | 11.26 |
| High | Low | -12.52 | 2.71 | ＜0.01** | -17.86 | -7.18 |
|  | Average | -8.09 | 1.61 | ＜0.01** | -11.26 | -4.92 |
| *P＜0.05, **P＜0.01. | | | | | | |

2.Supervisor's communication style with students

Method: LSD

| (I) Supervisor's communication style with students | (J) Supervisor's communication style with students | Mean Difference (I-J) | Std. error | P value | 95% Confidence interval | |
| --- | --- | --- | --- | --- | --- | --- |
|  |  |  |  |  | Lower bound | Upper bound |
| Tends to encourage | Tends to criticize | -14.47 | 5.69 | 0.01* | -25.68 | -3.26 |
|  | Tends to supervise | -3.76 | 1.61 | 0.02* | -6.93 | -0.59 |
| Tends to criticize | Tends to encourage | 14.47 | 5.69 | 0.01* | 3.26 | 25.68 |
|  | Tends to supervise | 10.71 | 5.77 | 0.07 | -0.65 | 22.07 |
| Tends to supervise | Tends to encourage | 3.76 | 1.61 | 0.02* | 0.59 | 6.93 |
|  | Tends to criticize | -10.71 | 5.77 | 0.07 | -22.07 | 0.65 |
| *P＜0.05, **P＜0.01. | | | | | | |

3.Age

Method: LSD

| (I) Age | (J) Age | Mean Difference (I-J) | Std. error | P value | 95% Confidence interval | |
| --- | --- | --- | --- | --- | --- | --- |
|  |  |  |  |  | Lower bound | Upper bound |
| 21-25 years old | 26-30 years old | 0.96 | 2.10 | 0.65 | -3.17 | 5.08 |
|  | 31-35 years old | 3.36 | 2.04 | 0.10 | -0.65 | 7.38 |
|  | ≥35 years old | 6.04 | 2.22 | ＜0.01** | 1.68 | 10.40 |
| 26-30 years old | 21-25 years old | -0.96 | 2.10 | 0.65 | -5.08 | 3.17 |
|  | 31-35 years old | 2.40 | 2.48 | 0.33 | -2.48 | 7.29 |
|  | ≥35 years old | 5.08 | 2.63 | 0.05 | -0.09 | 10.26 |
| 31-35 years old | 21-25 years old | -3.36 | 2.04 | 0.10 | -7.38 | 0.65 |
|  | 26-30 years old | -2.40 | 2.48 | 0.33 | -7.29 | 2.48 |
|  | ≥35 years old | 2.68 | 2.58 | 0.30 | -2.40 | 7.76 |
| ≥35 years old | 21-25 years old | -6.04 | 2.22 | ＜0.01** | -10.40 | -1.68 |
|  | 26-30 years old | -5.08 | 2.63 | 0.054 | -10.26 | 0.09 |
|  | 31-35 years old | -2.68 | 2.58 | 0.300 | -7.76 | 2.40 |
| *P＜0.05, **P＜0.01. | | | | | | |

4.Work experience

Method: LSD

| (I) Work experience | (J) Work experience | Mean Difference (I-J) | Std. error | P value | 95% Confidence interval | |
| --- | --- | --- | --- | --- | --- | --- |
|  |  |  |  |  | Lower bound | Upper bound |
| None | ≤2 years | 5.05 | 2.56 | 0.05* | 0.01 | 10.08 |
|  | 2-5 years | -3.23 | 3.67 | 0.38 | -10.45 | 4.00 |
|  | ≥5 years | 3.88 | 1.64 | 0.02* | 0.65 | 7.12 |
| ≤2 years | None | -5.05 | 2.56 | 0.05* | -10.08 | -0.01 |
|  | 2-5 years | -8.27 | 4.19 | 0.05* | -16.52 | -0.03 |
|  | ≥5 years | -1.17 | 2.60 | 0.66 | -6.28 | 3.95 |
| 2-5 years | None | 3.23 | 3.67 | 0.38 | -4.00 | 10.45 |
|  | ≤2 years | 8.27 | 4.19 | 0.05* | 0.03 | 16.52 |
|  | ≥5 years | 7.11 | 3.70 | 0.06 | -0.18 | 14.39 |
| ≥5 years | None | -3.88 | 1.64 | 0.02* | -7.12 | -0.65 |
|  | ≤2 years | 1.17 | 2.60 | 0.655 | -3.95 | 6.28 |
|  | 2-5 years | -7.11 | 3.70 | 0.056 | -14.39 | 0.18 |
| *P＜0.05, **P＜0.01. | | | | | | |

5.Reason for pursuing graduate studies

Method: LSD

| (I) Reason for pursuing graduate studies | (J) Reason for pursuing graduate studies | Mean Difference (I-J) | Std. error | P value | 95% Confidence interval | |
| --- | --- | --- | --- | --- | --- | --- |
|  |  |  |  |  | Lower bound | Upper bound |
| Voluntary | Employment or promotion pressure | -4.63 | 1.87 | 0.01* | -8.31 | -0.96 |
|  | Recommendation from others | -8.19 | 3.32 | 0.01* | -14.73 | -1.65 |
|  | Improve research skills | -1.70 | 2.00 | 0.40 | -5.64 | 2.24 |
| Employment or promotion pressure | Voluntary | 4.63 | 1.87 | 0.01* | 0.96 | 8.31 |
|  | Recommendation from others | -3.55 | 3.51 | 0.31 | -10.45 | 3.35 |
|  | Improve research skills | 2.93 | 2.29 | 0.20 | -1.58 | 7.45 |
| Recommendation from others | Voluntary | 8.19 | 3.32 | 0.01* | 1.65 | 14.73 |
|  | Employment or promotion pressure | 3.55 | 3.51 | 0.31 | -3.35 | 10.45 |
|  | Improve research skills | 6.49 | 3.58 | 0.07 | -0.56 | 13.53 |
| Improve research skills | Voluntary | 1.70 | 2.00 | 0.40 | -2.24 | 5.64 |
|  | Employment or promotion pressure | -2.93 | 2.29 | 0.201 | -7.45 | 1.58 |
|  | Recommendation from others | -6.49 | 3.58 | 0.071 | -13.53 | 0.56 |
| *P＜0.05, **P＜0.01. | | | | | | |
